# Plasma Membrane and Endomembrane Lipids Are Involved in a Complex Adaptation of *Arabidopsis thaliana* Hypocotyls to Cellulose Biosynthesis Inhibition

**DOI:** 10.3390/ijms27125424

**Published:** 2026-06-16

**Authors:** Ekaterina R. Kotlova, Svetlana V. Senik, Roman K. Puzanskiy, Gregory A. Pozhvanov, Oksana A. Rodina, Ekaterina M. Bogdanova, Bairta S. Manzhieva, Daria A. Frolova, Anna A. Manova, Dmitry V. Suslov

**Affiliations:** 1 Komarov Botanical Institute, Russian Academy of Sciences, 197022 St. Petersburg, Russia; senik@binran.ru (S.V.S.); puzansky@binran.ru (R.K.P.); pozhvanov@binran.ru (G.A.P.); orodina@binran.ru (O.A.R.); ebogdanova@binran.ru (E.M.B.); bmanzhieva@binran.ru (B.S.M.); 2 Department of Botany and Ecology, Faculty of Biology, Herzen State Pedagogical University, 191186 St. Petersburg, Russia; 3 Chemical Analysis and Materials Research Core Facility Center, Research Park, St. Petersburg State University, 199034 St. Petersburg, Russia; d.frolova@spbu.ru (D.A.F.); a.manova@spbu.ru (A.A.M.); 4 Department of Plant Physiology and Biochemistry, St. Petersburg State University, 199034 St. Petersburg, Russia

**Keywords:** plant lipids, lipidomics, phospholipids, galactolipids, lipid remodeling, cellulose, cellulose synthesis inhibitor, isoxaben, LC-QqQ-MS/MS, *Arabidopsis thaliana*

## Abstract

Cellulose is the strongest cell wall polymer defining plant cell shape and growth, and the most abundant biopolymer on the Earth. Its synthesis by the plasma membrane (PM)-localized cellulose synthase complexes (CSCs) depends on surrounding lipids that establish the membrane microenvironment in which CSCs work and form vesicles delivering and removing CSCs to and from the PM by exo- and endocytosis. The role of exact lipid molecular species in these processes is poorly understood. In the present work we used hypocotyls of etiolated wild-type Col-0 and mutant *ixr1-1 Arabidopsis thaliana* seedlings grown with or without isoxaben, a specific cellulose synthesis inhibitor, as a model to reveal lipid molecular species associated with cellulose biosynthesis. Different lipid classes were separated by thin-layer chromatography (TLC) and their molecular species were analyzed by liquid chromatography–triple quadrupole tandem mass spectrometry (LC-QqQ-MS/MS). A total of 250 lipid molecular species were identified. Col-0 plants maintained stable levels of membrane glycerophospholipids but displayed significant remodeling of their acyl chains. In the presence of isoxaben, they accumulated three times more phosphatidic acids, a hallmark of a stress response. The isoxaben-resistant mutant *ixr1-1* was characterized by a higher relative content of phosphatidylethanolamines, potentially rendering membranes more labile, as well as plastid galactolipids, which accumulated under isoxaben treatment. The multifaceted effects of isoxaben, including its impact on endomembrane lipids, suggest that it has additional binding sites beyond CSC.

## 1. Introduction

Membrane glycerophospholipids (GPLs) form the basis of the plasma membrane (PM) and endomembranes. Their diverse functions rely on molecular species heterogeneity determined by head groups, glycerol backbone linkage (acyl, alkyl, alkenyl), and fatty acid chain length/unsaturation [1,2]. This profile varies with taxonomy, developmental stages, cell types, and environmental conditions [3,4,5].

Two major classes of GPLs are phosphatidylcholines (PCs) and phosphatidylethanolamines (PEs). Both are highly heterogeneous, with 16:0 and 18:2 as the main acyl chains. In plants, PC and PE molecular species also often contain linolenic (18:3) acid. Cylinder-shaped PCs generally localize to the outer membrane leaflet, while conical PEs, associated with membrane curvature, typically occupy the inner leaflet of the plasma membrane [6,7]. The sn-1 fatty acid is typically saturated/monounsaturated, and the sn-2 acyl chain is usually polyunsaturated [2]. GPLs with saturated and long-chain fatty acids (16:0–18:0), including very-long-chain fatty acids (20:0–24:0, or sometimes longer), thicken and rigidify membranes, while unsaturated species increase their fluidity [2].

Minor GPL classes perform specialized functions. Phosphatidylinositols (PIs) prevailing on the inner PM leaflet are crucial for vesicular transport as precursors for phosphoinositides [8,9]. Phosphatidic acids (PAs) act as metabolic intermediates and second messengers, dynamically regulated by phospholipase D [10]. PA molecules predominantly accumulate on the inner leaflet of PM and in the endoplasmic reticulum (ER)—the major site of GPL biosynthesis. Phosphatidylserines (PSs), which are more predominant on the inner leaflet and enriched in very-long-chain fatty acids (VLCFAs), are a class of lipids important for ensuring interactions across the plasma membrane, as their aggregation provided by the actin cytoskeleton in the cytosolic leaflet causes clustering of glycosylphosphatidylinositol anchors in the outer leaflet [2]. As anionic lipids, PSs, PAs, and PIs determine membrane surface charge and modulate protein binding [11,12]. Their distinct shapes affect vesicle formation and protein recruitment: conical PIs promote positive curvature, while inverted-conical PSs/PAs induce negative curvature [13].

GPL composition defines organelle identity. The ER, enriched with unsaturated PCs/PEs, forms thin, fluid membranes, the PM is thicker and more rigid, while Golgi exhibits intermediate properties [7]. Phosphatidylglycerols (PGs) occur in plastids and mitochondria; diphosphatidylglycerols (DPGs) (cardiolipins) are mitochondrial-specific. Unlike most endomembranes, thylakoid membranes are primarily composed of non-phosphorus galactoglycerolipids (GGLs), specifically monogalactosyldiacylglycerols—(MGDGs) and digalactosyldiacylglycerols (DGDGs). These GGLs make up approximately 80% of thylakoid lipids, with the remaining 20% consisting of sulfoquinovosyldiacylglycerols (SQDGs) and PGs [14]. In contrast, the outer chloroplast envelope has a distinct composition: in addition to GGLs, SQDGs, and PGs, approximately 30% is represented by PCs [15]. The asymmetric distribution of lipids across organelles and between the leaflets of the PM and endomembranes is maintained by the concerted action of biosynthetic enzymes, flippases, lipid transfer proteins, and vesicular transport [16,17]. This distribution is further reinforced by the intrinsic self-organization of lipid molecules based on their specific structural properties [6,7].

PM GPLs are part of a surface continuum, contacting the cytoskeleton internally and cellulose microfibrils externally [9]. Cellulose microfibrils, synthesized by PM-localized cellulose synthase complexes (CSCs), are the strongest cell wall polymers that define cell shape and growth direction [18]. CSCs are assembled at the Golgi [19] and delivered to the PM in vesicles [20]. Once at the PM, CSCs are activated and start producing cellulose chains extracellularly using cytoplasmic UDP-glucose as a precursor [21]. These emerging microfibrils interact with preexisting cell wall components and become immobilized within the wall. Nascent cellulose microfibrils fixed at one end within the wall and growing at the opposite end by CSC synthetic activity push the CSCs such that they float along the PM at a velocity proportional to the efficiency of cellulose synthesis [22]. Detailed analyses have demonstrated that cellulose polymerization rate (the number of glucose residues added to the growing cellulose chain per time unit) was limited by microfibril crystallization (the rate at which new cellulose chains are assembled into a paracrystalline microfibril) [23]. Hence, CSC velocity at the PM is defined by the microfibril crystallization process. CSCs typically reside at the PM producing cellulose for 10 min, after which they are internalized to the cortical layer of cytoplasm by endocytosis accumulating in vesicular structures called small CESA compartments or microtubule-associated CESA compartments followed by their recycling to the PM or degradation [21]. These processes—exocytosis, catalytic activity, endocytosis—are likely influenced by membrane lipids, yet direct evidence is scarce. Sterol and sphingolipid mutants/inhibitors caused cellulose deficiency, implying the involvement of lipids in microfibril biosynthesis and deposition [24,25]. Patatin-related phospholipases affected cellulose content and cell growth, suggesting broader links between lipid metabolism, carbon partitioning, and cytoskeletal dynamics [26,27,28].

Isoxaben, a specific cellulose synthesis inhibitor, blocks UDP–glucose incorporation into nascent glucan chains. Molecular docking analysis predicted two different isoxaben binding sites at the transmembrane regions of a CSC subunit close to the extracellular side of the PM and the cellulose-conducting channel adjacent to the growing glucan chain [29]. On the other hand, indirect data suggests that isoxaben could have binding sites beyond CSCs as well [30]. This inhibitor rapidly cleared CSCs from the PM resulting in their accumulation in vesicles below the membrane [31]. It also decreased CSCs velocity at the PM [32]. These isoxaben effects could be partially mediated by membrane lipids affecting endocytosis and membrane fluidity, respectively.

The point mutation *ixr1-1* was first identified in a screen for *A. thaliana* lines resistant to isoxaben [33]. The isoxaben resistance in *ixr1-1* is conferred by a glycine to aspartic acid replacement at one of the transmembrane domains of a CSC subunit [34]. Until recently [30], the resistance was explained by a conformational change that prevented isoxaben binding to the CSC subunit. This mutation also reduced cellulose crystallinity but did not change its total content in etiolated *ixr1-1 Arabidopsis* seedlings compared with Col-0 [35]. Interestingly, reduced cellulose crystallinity in a closely related *ixr1-2* mutant background was associated with higher CSCs velocities in *Arabidopsis* hypocotyls [23].

Our work aimed to identify lipid molecular species associated with cellulose synthesis. We used hypocotyls of etiolated wild-type Col-0 and mutant *ixr1-1 Arabidopsis thaliana* seedlings grown with or without isoxaben as a model. According to the literature, these variants have contrasting CSC velocities in the PM and different CSC internalization rates, which may highlight different aspects of lipid involvement in the process of cellulose biosynthesis. Hypocotyls of etiolated *A. thaliana* seedlings have long been used as a model to study the mechanisms of plant cell diffuse growth. Cell divisions contributing to this juvenile organ formation are essentially restricted to the stage of hypocotyl establishment inside the seed. Its emergence during seed germination and subsequent elongation are entirely mediated by cell expansive growth without accompanying divisions [36]. Cell growth is slow along the hypocotyl axis during the first two days after seed imbibition, after which a wave of rapid cell elongation is initiated at the base of hypocotyls and spreads towards cotyledons [37]. Hypocotyls of four-day-old etiolated Col-0 seedlings used in the present study contain growing cells in the upper half of this organ and completely elongated cells in its lower half [36]. Growth of entire hypocotyls starts decelerating in four-day-old Col-0 seedlings [38], and their use in the present study is a compromise between the need to study a growing tissue and the ability to collect a sufficient amount of plant material for lipidomics.

We have demonstrated that in wild-type Col-0 plants, the level of membrane GPLs remained sufficiently stable at the class level but displayed significant remodeling of acyl chains that included a rise in PC and PA unsaturation and an increased proportion of PE and PS species containing very-long-chain fatty acids (VLCFAs). Elevated PA levels accumulated in Col-0 grown with isoxaben highlight the involvement of PA signaling related to a stress response. The *ixr1-1* mutant exhibited two distinct lipid changes associated with isoxaben resistance: (1) the accumulation of PEs, with a higher representation of atypical molecular species (odd-chain species, highly asymmetric chain-length species), which potentially increases membrane fluidity and disorder, rendering them more labile; and (2) the accumulation of plastid galactolipids MGDG and DGDG, which may integrate into other membranes, including the PM, indicated by changes in their acyl moieties, which are partially replaced by those characteristic of GPLs. The systemic response at the lipidome level suggests that isoxaben has additional binding sites beyond those located on CSCs.

## 2. Results

### 2.1. Ultra-Low Concentration of Isoxaben Suppresses Growth in Wild-Type Col-0 Plants but Not in ixr1-1 Mutants

**Figure 1:**
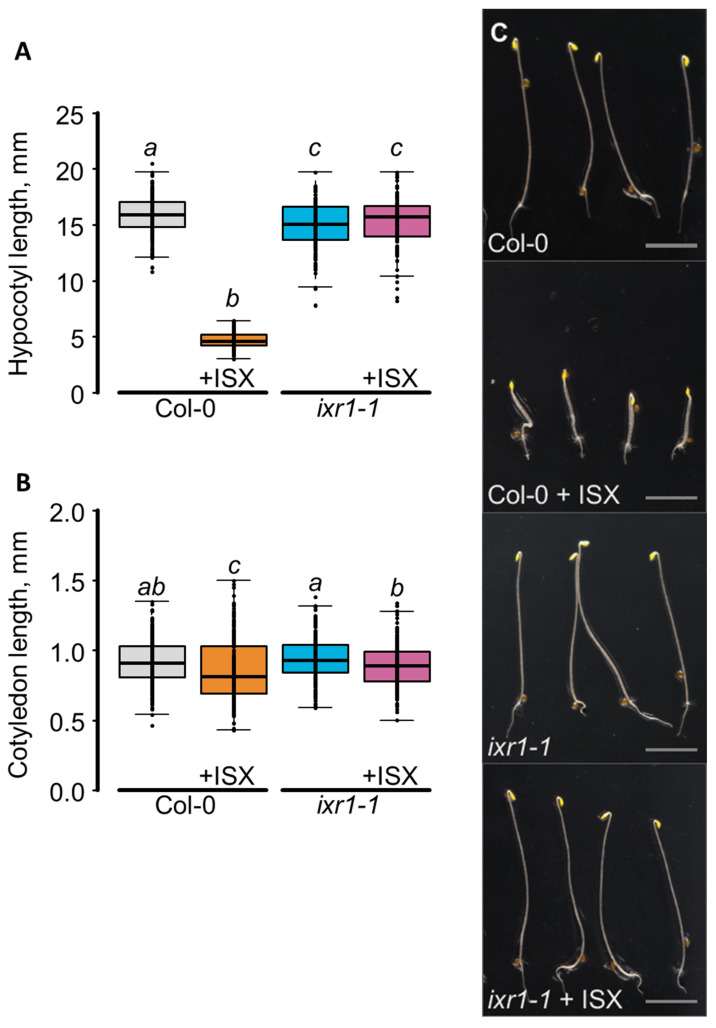
Growth and morphology of etiolated wild-type (Col-0) and *ixr1-1* mutant *A. thaliana* seedlings grown on the medium with or without 0.2 nM isoxaben. Data are represented as a box from the 1st to the 3rd quartile with a marked median. The same lower case letters in italics mark values for groups that do not differ significantly; values that do not share the same letters differ significantly (*p* < 0.05; adjusted for multiple comparisons *p*-values from Mann–Whitney–Wilcoxon rank test; *n* = 40–74 from three independent experiments). (**A**)—hypocotyl length of 4-day-old etiolated *A. thaliana* seedlings grown on one-half-strength Murashige–Skoog medium without sucrose in the absence (no mark) and presence (+ISX) of 0.2 nM isoxaben at 21 °C in darkness. (**B**)—cotyledon length of 4-day-old etiolated *A. thaliana* seedlings. (**C**)—typical 4-day-old etiolated wild-type (Col-0) and *ixr1-1* mutant seedlings of *A. thaliana* grown with (+ISX) or without 0.2 nM isoxaben. Scale bars are 5 mm.

Four-day-old wild-type (Col-0) and *ixr1-1* mutant plants of *A. thaliana* grown in darkness on one-half-strength MS medium without sucrose exhibited typical phenotype for etiolated seedlings with long hypocotyls and yellowish cotyledons bent downwards. Growing them in the presence of isoxaben (0.2 nM) in the medium resulted in substantial alterations in plant growth and morphology. This cellulose biosynthesis inhibitor significantly decreased hypocotyl growth in Col-0 by 70%, but not in *ixr1-1* (Figure 1A,C). Cotyledons of wild-type seedlings grown in the presence of isoxaben were 6% shorter compared with untreated Col-0 (Figure 1B). We also observed a weak but significant growth reduction of *ixr1-1* vs. Col-0 hypocotyls in the absence of isoxaben (Figure 1A).

### 2.2. Col-0 and ixr1-1 Plants Exhibit Distinct Glycerolipid Class Ratios and Contrasting Responses to Isoxaben Treatment

**Figure 2:**
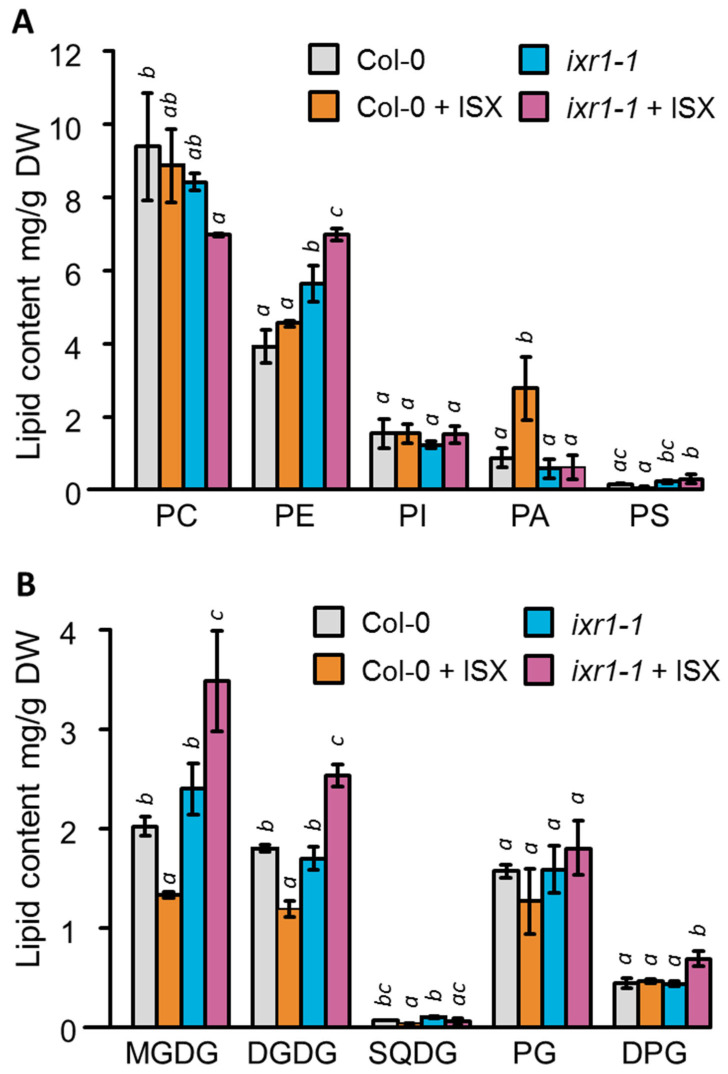
Profiling of glycerolipid classes of 4-day-old etiolated wild-type (Col-0) and *ixr1-1* mutant seedlings of *A. thaliana* grown on the medium with and without 0.2 nM isoxaben. (**A**) Phospholipids constituting the plasma membrane and endomembranes (intracellular organelle membranes). (**B**) Glyco- and phospholipids constituting the endomembranes (intracellular membranes of organelles, primarily plastids and mitochondria). Data are means ± SD (*n* = 3). The same letters indicate values that do not differ significantly and belong to the same group; values that do not share the same letters differ significantly (one-way ANOVA with Tukey post hoc test, *p* < 0.05). PC—phosphatidylcholines; PE—phosphatidylethanolamines; PI—phosphatidylinositols; PA—phosphatidic acids; PS—phosphatidylserines, MGDG—monogalactosyldiacylglycerols, DGDG—digalactosyldiacylglycerols, SQDG—sulfoquinovosyldiacylglycerols, PG—phosphatidylglycerols, DPG—diphosphatidylglycerols (cardiolipins).

PCs, PEs, PIs, PAs, and PSs were found among the GPLs classes, which are the basic components of the PM and secretory pathway endomembranes including ER and Golgi, in 4-day-old etiolated *A. thaliana* seedlings. The absolute lipid content in these classes was similar in wild-type Col-0 and *ixr1-1* mutant plants with the exception of PEs, the content of which was 44% higher in the mutant than in the wild type (Figure 2A). As a result, the PC/PE ratios were 2.3 and 1.5 in the wild type and the mutant, respectively.

Isoxaben did not change the content of PCs and PEs in Col-0 cell membranes. On the contrary, *ixr1-1* grown with isoxaben demonstrated a trend toward a decreased PC content, compensated by a significantly increased PE content, which leads to a reduction in the PC/PE ratio to 1.0. Isoxaben also affected the content of minor lipid classes in the wild type. In particular, it resulted in a 3-fold accumulation of PAs. No significant isoxaben-induced differences in the minor GPLs were detected in the mutant (Figure 2A).

Cell organelle lipids, including those of plastid and mitochondrial origin, were also analyzed, although they are not directly involved in cellulose synthesis and CSC catalytic activity. The content of plastid (etioplast)-associated lipids MGDGs and DGDGs was similar in the both genotypes, and their ratios were 1.1 and 1.4 for Col-0 and *ixr1-1*, respectively.

Isoxaben had a different effect on GGLs in Col-0 and *ixr1-1* plants (Figure 2B). In the wild type, it induced a consistent decrease in the content of MGDGs and DGDGs. On the contrary, the content of MGDGs and DGDGs increased by about 40% for each of the two lipid classes in the mutant. In contrast to GGLs, the content of SQDGs, a class associated with maintaining thylakoid membrane structure, decreased upon incubation with isoxaben in both Col-0 and *ixr1-1*.

A stable level of PGs, another class of endomembrane lipids with specific functions that, in addition to plastids, is also found in mitochondria, was characteristic for all samples regardless of genotype. In contrast, the content of DPGs, typical for mitochondrial membranes, remained unchanged in Col-0 but increased in the mutant upon isoxaben treatment. Significant and opposite effects of isoxaben on MGDG and DGDG content in Col-0 and *ixr1-1* etioplasts, as well as differential responses of the mitochondrial marker DPGs, indicate a complex interplay between cellulose synthesis, lipid homeostasis under isoxaben treatment, and endomembrane functions in these processes.

### 2.3. Differences Between Col-0 and ixr1-1 at the Level of Major GPL and GGL Molecular Species Are Prominent Only Under Isoxaben Treatment

**Figure 3:**
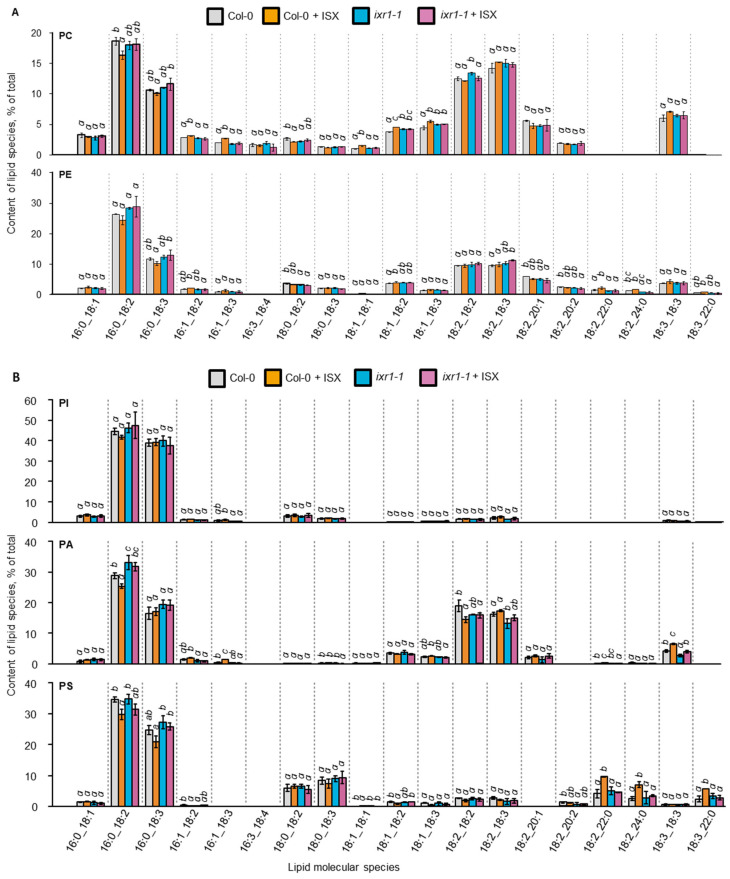
Profiling of phospholipid molecular species in 4-day-old etiolated wild-type (Col-0) and *ixr1-1* mutant seedlings of *A. thaliana* grown on the medium with or without 0.2 nM isoxaben. (**A**) Major phospholipids of the plasma membrane and endomembranes (organelle membranes). (**B**) Minor phospholipids of the plasma membrane and endomembranes (organelle membranes). PC—phosphatidylcholines, PE—phosphatidylethanolamines, PI—phosphatidylinositols, PA—phosphatidic acids, PS—phosphatidylserines. Data are means ± SD (*n* = 3). The same letters indicate values that do not differ significantly and belong to the same group; values that do not share the same letters differ significantly (one-way ANOVA with Tukey post hoc test, *p* < 0.05). Note that the stereospecific position of each acyl group on the glycerol backbone could not be assigned.

**Figure 4:**
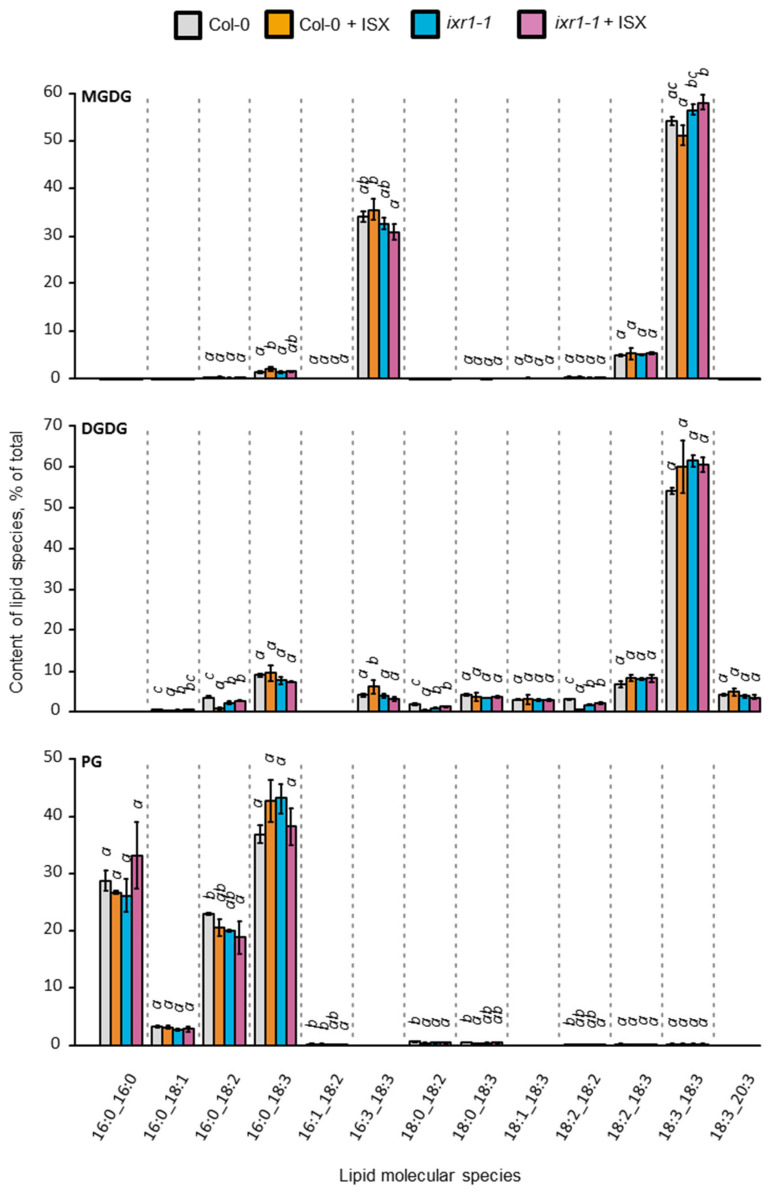
Profiling of glyco- and phospholipid molecular species of endomembranes (organelle membranes) in 4-day-old etiolated wild-type (Col-0) and *ixr1-1* mutant seedlings of *A. thaliana* grown on the medium with or without 0.2 nM isoxaben. MGDG—monogalactosyldiacylglycerols, DGDG—digalactosyldiacylglycerols, PG—phosphatidylglycerols. Data are means ± SD (*n* = 3). The same letters indicate values that do not differ significantly and belong to the same group; values that do not share the same letters differ significantly (one-way ANOVA with Tukey post hoc test, *p* < 0.05). Note that the stereospecific position of each acyl group on the glycerol backbone could not be assigned.

Molecular profiles of basic GPL and GGL classes in Col-0 and *ixr1-1* plants grown with or without isoxaben were studied using LC-QqQ-MS/MS. In order to distinguish changes in general physico-chemical properties of membranes from more subtle changes associated with integral proteins including CSCs, a functional group describing membrane state as a matrix was selected from a general lipid profile. This group included major molecular species, each of which accounted for ≥1% of the sum of all molecular species identified in a class. Their ratio defines basic physico-chemical membrane characteristics (fluidity, packing density, thickness, surface charge). To estimate the contribution of these species to the bilayer properties, their content was expressed as a percentage of the total pool of a class. Figure 3 shows the profiles of GPLs that form the PM and secretory pathway endomembranes, including major (Figure 3A) and minor (Figure 3B) classes. Figure 4 describes molecular profiles of basic lipid classes associated with etioplast membranes.

Four molecular species prevailed in the profile of PCs with the content of each of them exceeding 10%: two species with mixed-length 16/18 chains, including 16:0_18:2 and 16:0_18:3, and two species with 18/18 chains, including 18:2_18:2 and 18:2_18:3. Among all the GPL classes analyzed, PCs demonstrated the maximum content of polyunsaturated molecular species with linolenic C18:3 fatty acid, such as 18:1_18:3 and 18:3_18:3, as well as a marker 16:3_18:4 found in PCs only. A similar distribution of molecular species was found in PAs.

Col-0 and *ixr1-1* plants demonstrated almost complete similarity in the composition of the main molecular species for PCs and PAs (Figure 3A,B). However, when treated with isoxaben, the response at the level of the main molecular species was different. While *ixr1-1* maintained homeostasis in the distribution of major PC and PA molecular species, a slight substitution was observed in Col-0. In particular, in the PC profile, 16:0_18:2 and 18:0_18:2 were partially substituted by 16:1_18:2, 16:1_18:3, 18:1_18:2, and 18:1_18:3. In the PA profile, a substitution of 16:0_18:2 by 16:1_18:3 was combined with a substitution of 18:2_18:2 by 18:3_18:3. This practically synchronous increase in the unsaturation of PC and PA molecular species indicates the activation of desaturation reactions directly on PC molecules, some of which serve as precursors in the synthesis of PAs.

The molecular profile of PEs was characterized by a higher relative content of mixed-chain molecular species, with a clear dominance of 16:0_18:2 accounting for more than 25%. The profile of this class also demonstrated a prominent contribution of molecular species with very-long-chain fatty acids (VLCFAs), such as 18:2_20:1, 18:2_20:2, 18:2_22:0, 18:2_24:0, totaling more than 15%. PS profiles had a certain similarity to those of PEs. However, in the PS profile, in contrast to PEs, the content of molecular species with 16:0 and 18:0 was more than 70%, while in PEs it was about 50%. Another feature of PSs was the distribution pattern of VLCFAs, with a predominance of molecules having longer chains, such as 22:0 and 24:0.

Col-0 and *ixr1-1* plants were very similar in the composition of their PE and PS molecular species; however, the response of the two genotypes to isoxaben treatment was again different. The mutant maintained homeostasis at the level of molecular species. On the contrary, Col-0 demonstrated a pronounced decrease in the relative content of 16:0_18:2 in the PS profile, which was compensated by a 2-3-fold increase in the contribution of very-long-chain molecular species: 18:2_22:0, 18:3_22:0 and 18:2_24:0. The differences between the genotypes in the PI molecular profiles, as well as lipids associated with plastid membranes, turned out to be minimal. The response to isoxaben at the level of the main molecular species was also insignificant in these classes.

Thus, analysis of the major molecular species revealed that *ixr1-1* maintained phospholipid stability during incubation with isoxaben, whereas in Col-0 the inhibitor induced lipid remodeling. These changes in the wild type were expressed as an increase in acyl chain unsaturation in PCs and PAs and a simultaneous increase in the proportion of VLCFAs in PSs, which together may result in altered membrane physicochemical properties. The greatest plasticity in response to isoxaben treatment was found in PCs, PAs, and PSs, whereas the profiles of PIs and plastid lipids remained highly conserved in both genotypes.

### 2.4. Complex Profiling of GPLs and GGLs, Including Minor Molecular Species, in Col-0 and ixr1-1 Reveals Genotype-Specific Differences Increased by Isoxaben

Minor molecular species (<1% within each lipid class) are difficult to analyze because of their abundance, diversity, and technical limitations. To address this, we combined preparative TLC for lipid class isolation with LC-QqQ-MS/MS for separation and identification of molecular species. Using this approach, we identified 250 lipid molecular species. The greatest diversity was observed among major GPLs, including PCs (61 species) and PEs (74 species). Minor GPL classes contained fewer species: PAs (28), PIs (15), and PSs (15). Etioplast GGLs comprised 18 MGDG and 19 DGDG species, whereas 20 PG species were detected. Individual sample data are provided in Table S1 (Supplementary Material). Total lipid profiles are presented as absolute values per gram of dry weight to reflect actual quantitative changes.

**Figure 5:**
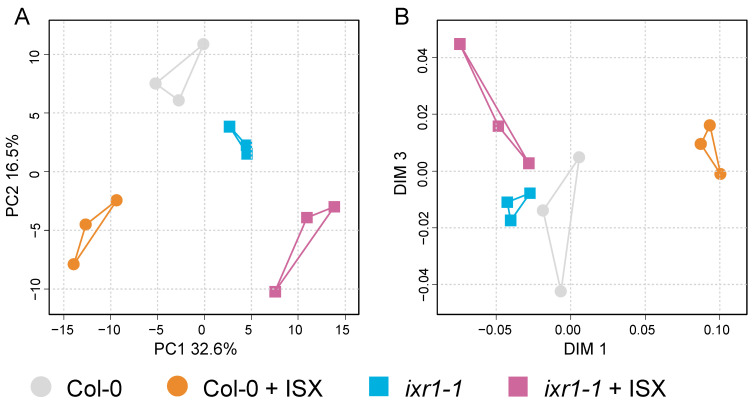
Representation of lipid profiles of 4-day-old hypocotyls of wild-type and *ixr1-1 A. thaliana* mutants in low dimensional spaces. (**A**)—Principal component analysis (PCA) score plots. (**B**)—Scattering in the coordinates extracted by multidimensional scaling (MDS) using (1–rho) as a distance measure, where rho is the Spearman’s correlation coefficient. Colored circles and squares correspond to the particular lipid profiles, % is the percentage of variance related to the corresponding principal component (PC1, PC2).

To visualize overall lipidome variation, we first applied principal component analysis (PCA) (Figure 5A). Lipid profiles clustered according to genotype and isoxaben treatment. Differences between Col-0 and *ixr1-1* were mainly associated with a principal component 1 (PC1, 32.6%), whereas the effect of isoxaben corresponded to a principal component 2 (PC2, 16.5%). The effect was more pronounced in Col-0.

Because non-parametric distance measures are less sensitive to normalization procedures, we additionally used 1 − r as a distance metric, where r is Spearman’s rank correlation coefficient between lipid profiles. Multidimensional scaling (MDS) based on these distances confirmed genotype-dependent differences that became stronger after isoxaben treatment (Figure 5B). These effects were especially prominent when the complete profiles, including minor molecular species, were analyzed.

A comparison of selected groups of molecular species revealed several subtle but consistent genotype-specific differences that became more pronounced under isoxaben treatment. Col-0 contained more mixed-chain PC species with one saturated and one monounsaturated fatty acid, whereas *ixr1-1* accumulated more PE species containing one saturated and one polyunsaturated fatty acid. In addition, PE species with odd-chain fatty acids (e.g., 15:0, 17:0, 17:1, 19:1, 21:0, 23:0) increased in *ixr1-1* under isoxaben treatment but not in Col-0.

**Figure 6:**
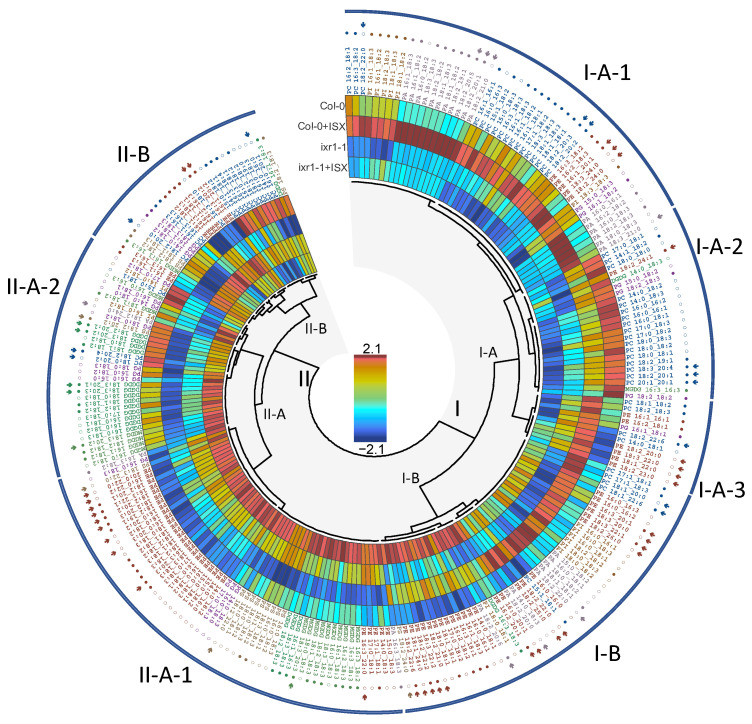
Heatmap of the lipid molecular species content. Data were standardized. Lipids were clustered using Spearman’s correlation and Ward’s method. Arrows indicate molecular species containing very-long-chain fatty acids (VLCFAs). Filled circles indicate molecular species containing two unsaturated fatty acids; open circles indicate molecular species containing one or two saturated fatty acids. The lipid molecular species names and the symbols denoting their classification into groups containing VLCFAs, saturated, or unsaturated fatty acids are color-coded according to their respective lipid class.

To identify co-varying molecular species, we performed hierarchical clustering based on Spearman’s rank correlation. The resulting heat map and dendrogram revealed two major clusters (Figure 6). Notably, this clustering was driven by the response of Col-0 lipids to isoxaben, despite the inclusion of all four experimental groups (Col-0, Col-0 + ISX, *ixr1-1*, *ixr1-1* + ISX). Cluster I contained lipids whose levels increased or remained high in Col-0 under isoxaben treatment, whereas Cluster II contained lipids whose levels decreased or remained low in the presence of the inhibitor but increased in *ixr1-1*.

Subcluster I-A contained species with higher levels in Col-0 than in *ixr1-1*. Subcluster I-A-1 included species increased by isoxaben in Col-0, while a similar but more pronounced pattern (a stronger increase) characterized subcluster I-A-3. These subclusters were distinguished by the presence of highly unsaturated PC species with two unsaturated fatty acids. Subcluster I-A-2, which included molecular species with maximal accumulation in untreated Col-0 plants, was primarily composed of PCs characterized by varying acyl chain lengths, one of which was saturated. Subcluster I-B comprised species that accumulated upon isoxaben treatment regardless of genotype, including many PE species with VLCFAs, PS 18:2_24:0, as well as several PI and PA species with one saturated and one unsaturated fatty acid.

Cluster II mainly included molecular species that were more abundant in the *ixr1-1* mutant (treated or untreated with isoxaben) than in Col-0. Subclusters II-A-1 and II-A-2 combined lipids whose levels further increased in *ixr1-1* upon isoxaben treatment. They contained many MGDGs and DGDGs, as well as a significant proportion of mixed-chain PE and PS species with one saturated and one unsaturated fatty acid. The presence of PE and PS pairs with identical acyl chains (e.g., PE 16:0_18:3 and PS 16:0_18:3) suggests an active conversion of PE to PS in the mutant during isoxaben treatment. Subcluster II-B, found at the bottom of the heat map, was of particular interest. It included molecular species whose levels were higher in *ixr1-1* in the absence of isoxaben but decreased upon the inhibitor treatment. This “steady-state lipidome” [39] included major PC species, such as PC 16:0_18:2 and PC 18:2_18:2, along with unsaturated PE and PS species. It was significantly enriched with 16/16 and 16/18 molecular species, including PC 16:3_18:4.

To associate lipid species with genotype and isoxaben treatment, we performed set enrichment analysis (SEA) using factor loadings from OPLS-DA models. The results are shown in Figure S1 as normalized enrichment scores (NESs) and log10(q). Col-0 was characterized mainly by mixed-chain DGDG and PC species containing one saturated and one unsaturated fatty acid. In contrast, *ixr1-1* was distinguished by higher levels of PE and, to a lesser extent, PS species, many of which also contained mixed-chain acyl groups with a saturated fatty acid. Isoxaben treatment in Col-0 was associated with increased levels of PA species, unsaturated PCs, and saturated PEs. In *ixr1-1* isoxaben promoted the accumulation of MGDG and DGDG species, as well as saturated and odd-chain PE, PA, and PI species, including PE 15:0_18:1 and PE 15:0_18:3.

Thus, our lipidome analysis has revealed two antagonistic lipid modules: (i) a cellulose inhibition-induced cluster enriched with unsaturated PCs, PAs, and VLCFA-containing PEs, and (ii) a cluster associated with adaptation and the normal state, enriched with mixed-chain PEs with saturated fatty acids, 16/18 PCs, and etioplast lipids.

## 3. Discussion

### 3.1. Basic Mechanisms by Which Lipids Could Affect Cellulose Synthesis

Direct effects of GPLs on membrane proteins, including CSCs, arise from their ability to modulate membrane physico-chemical properties–fluidity, thickness, packing density, curvature, tension, and electrostatics (collectively referred to as the matrix effect) [40]—as well as to influence protein conformation and oligomerization (stereospecific effect) [41]. Due to these factors, the observed lipid modifications may influence CSC mobility in the PM and their catalytic activity. GPL involvement is also important for CSC synthesis on ER membranes, their processing at Golgi, subsequent delivery to the PM, and renewal by endocytosis. Besides their transport role, the inner leaflet lipids, as a part of the peripheral continuum, along with specific proteins, may mediate the link of CSCs with microtubules, affecting their lateral and vertical mobility as well as substrate linkage. The outer leaflet lipids may participate in mechanoperception reactions that perceive and transmit signals from an event in the cell wall to the PM and CSC.

Changes in the whole lipidome, as well as in the lipid profiles of cell organelle endomembranes (plastids, mitochondria), characterize the systemic cell response to cellulose synthesis inhibition, as well as adaptive reactions that protect the cell with damaged cell walls from irreversible defects. As a part of the remodeling process involving numerous phospholipases (A-, C-, D-types), lipids can also participate in the redistribution of carbon fluxes for cellulose synthesis [26]. Lipid conversion into sugars is carried out via glyoxysomes, the role of which is highly important during seed germination and early seedling development. This process is facilitated by contacts between the PM, ER and a plastid (etioplast) [42].

### 3.2. Growth Effects of Isoxaben and the ixr1-1 Mutation

The growth inhibition in the presence of isoxaben (Figure 1A) is known to result from cancelling the wave of rapid cell elongation from the base of hypocotyls towards cotyledons [37]. As plant cell growth is limited by cell wall extensibility at the biophysical level [43] and cellulose is the strongest and the least extensible cell wall component [44], it looks counterintuitive that the reduction of cellulose biosynthesis by isoxaben inhibits growth in *A. thaliana* hypocotyls. In fact, this unexpected growth response is caused by the activation of cell wall integrity maintenance mechanisms in the presence of isoxaben [45]. Plant cells are able to monitor the functional integrity of their cell walls and initiate adaptive responses including growth inhibition if the wall integrity is impaired. They perceive the isoxaben-induced cellulose biosynthesis inhibition as a cell wall damage and stress, arresting their elongation to prevent the wall failure. Interestingly, isoxaben was one of the tools that helped identify the existence of cell wall integrity sensing in plants [46] and the role of receptor-like kinases in this process [47]. Thus, some lipid modifications reported in the present work could be associated not only with changes in cellulose biosynthesis but also with the more general mechanisms of stress response and cell wall integrity maintenance.

The weak but significant growth inhibition of *ixr1-1* vs. Col-0 hypocotyls in the absence of isoxaben (Figure 1A) was consistent with earlier findings. Previous studies have demonstrated contradictory effects of *ixr1-1* on hypocotyl elongation in comparison with Col-0 plants: an obvious decrease or no effect on growth in different experiments of the same work [48], a statistically significant inhibition [35] or a slight growth reduction, although insignificant [49]. Thus, the effect of *ixr1-1* on hypocotyl elongation apparently depends on fine differences in growing conditions of *A. thaliana* seedlings.

### 3.3. Isoxaben Has Different Targets in Plant Cells

Isoxaben has traditionally been viewed as a specific CSC inhibitor, but its action is considerably more complex. The simplest model of isoxaben resistance conferred by the *ixr1-1* mutation and similar point mutations postulates that the inhibitor has a specific binding site on one of the CSC subunits, and a conformational change in the CSC resulting from a single amino acid substitution interferes with isoxaben binding to its target site. Indeed, molecular modeling predicted isoxaben-binding sites on the CSC [29]. However, several lines of evidence challenge this simplistic view. First, some cellulose biosynthesis inhibitor-resistant mutations affect proteins that are not part of the CSC, e.g., those of mitochondrial localization [50,51]. Second, the growth effect of isoxaben was completely different in *A. thaliana* shoots and roots, despite the fact that the same CSCs work in both organs and the inhibitor is easily transported and not metabolized in them [30]. The results of our work, namely, the significant differences in the content of certain lipid classes between *ixr1-1* plants treated and untreated with isoxaben (Figure 2A,B), also support a non-straightforward mechanism of CSC inhibition by isoxaben. At the same time, the high lipid molecular species homeostasis observed in *ixr1-1* irrespective of the presence of isoxaben (Figure 3A,B; Figure 4) suggests that this aspect of lipid modulation by the inhibitor may be mediated by a direct isoxaben–CSC interaction. It is possible that isoxaben acts not on the CSC protein alone but on the entire lipid–protein complex. Apparently, there are not only additional binding sites for isoxaben distinct from CSCs, but also parallel regulatory circuits associated with them. For example, isoxaben-induced cell death in *A. thaliana* cell suspension cultures was repressed by inhibitors blocking calcium influx and calcium release from internal stores [52], indicating that cytosolic calcium changes are an early step in the signaling cascade triggered by isoxaben. It was also shown that low concentrations of nitrate (6 mM) abolished isoxaben effects on growth in *A. thaliana* roots [30]. Nitrates are easily reduced to nitrogen oxide, a potent gas transmitter that acts as an intermediate in auxin-dependent regulation [53] involving auxin response factors and auxin receptors [54]. Auxin is presumably linked with CSCs via PIN transporters that are localized in the vicinity of CSCs—possibly in the same or adjacent lipid microdomains—and lose their normal localization under isoxaben action, separating from CSCs [52]. Since auxin can upregulate PIN expression and restore their polarized localization, auxin treatment could potentially reestablish CSC–PIN complexes that are degraded due to isoxaben-induced cell wall defects.

Taken together, these data suggest that isoxaben may have different targets in a plant cell, including CSC itself and possibly a lipid–protein complex or an entire microdomain that includes, in addition to CSC, various lipids and proteins responsible, on the one hand, for CSC–cytoskeleton interactions and, on the other hand, for interactions with the cell wall. This complexity can be successfully captured in our experimental model, where rapid elongation is coupled with continuous cellulose deposition and membrane assembly. These processes involve tight coordination between CSC activity and lipid asymmetry resulting from vesicular transport, lipid metabolism and their dynamic remodeling. Our model provides an ideal context to observe the functional crosstalk between the lipid environment and the CSC machinery.

### 3.4. Wild-Type Col-0 Strategy: Fine-Tuned Remodeling and Stress Response

Col-0 responds to isoxaben with fine lipid remodeling at the molecular species level, which is absent in the mutant (Figure 3). Thus, this aspect of lipid metabolism regulation could be mediated by isoxaben binding to its site on the CSC, which is impaired in *ixr1-1*.

The wild-type response (Figure 3A,B and Figure 6, Subcluster I-A) involved a decrease in the content of saturated PC molecular species and an increase in the concentration of unsaturated PCs together with their complementary unsaturated PAs, indicating the involvement of PA signaling. The presence of similar response patterns to isoxaben in PCs and PAs may indicate a high level of PCs recycling via PAs without acyl residue remodeling by phospholipase D or unspecific phospholipase C together with diacylglycerol kinase [1,55]. The similarity of PC and PA molecular profiles has been repeatedly reported, including for *A. thaliana*. This trend persisted at different plant developmental stages and during the diurnal dark–light cycle, where 16:0_18:3, 18:2_18:2 and 18:2_18:3 accumulated more actively at the end of the dark period [1,56]. The high degree of unsaturation of the PC and PA profiles found in our study, in particular the accumulation of PC18:3_18:3, PA18:3_18:3, and PC16:3_18:4, may also be typical for membranes of etiolated hypocotyls. PA is a well-known stress marker. In Col-0, the total PA content demonstrated a 3-fold increase upon isoxaben treatment (Figure 2A)—the clearest evidence of stress conditions among all our data. This 3-fold PA accumulation occurred unevenly. On the background of decreased relative content of PA 16:0_18:2 and PA 18:2_18:2, the content of PA 18:3_18:3 increased sharply (Figure 3B), along with a number of other molecular species containing 18:3 (Figure 6). PA accumulation enhances the plant’s ability for transmembrane transport and signal transmission [57] via multiple PA-binding proteins, including protein kinases, protein phosphatases, transcriptional factors, and proteins involved in endocytosis or cytoskeleton formation.

Changing the PC profile can affect not only membrane physico-chemical properties, but it is also of great importance for the cell as a whole. PC serves as the main acyl hub in plants, being the predominant phosphoglyceride lipid in most plant cells. PC plays a central role in several aspects of plant lipid metabolism, acting as the main substrate for extraplastid modifications of acyl chains, in lipid transport between subcellular compartments, and, through the remodeling process, in triacylglycerol synthesis [58,59]. Depending on the structure of their fatty acid acyl chains, PCs can affect plant growth and morphology. This effect was demonstrated on *A. thaliana* in experiments with exogenous PCs: PC16:0_16:0 and PC16:0_18:1 elongated seedling roots, whereas PC16:0_18:2 and PC18:2_18:2 shortened them [60]. A study of GPL molecular species distribution in young, actively growing and old leaves of *A. thaliana* also demonstrated the relationship between structure and growth [5]. The levels of PC16:3_18:3 and PE16:3_18:3 were significantly higher in more mature leaves than in young ones; for PC16:0_18:1 and PE16:0_18:1, the opposite trend was observed. An increase in PC unsaturation in Col-0 in response to isoxaben on the background of growth inhibition may be another indirect evidence for the ability of various PC molecular species to activate growth and the mechanisms of developmental regulation.

In addition to PCs and PAs, PSs were also involved in the Col-0 response to isoxaben. While their total content did not change significantly, substantial changes in the profile of their major molecular species were revealed. In particular, the content of VLCFA-containing species, including PS 18:2_22:0, PS 18:3_22:0, and PS 18:2_24:0, demonstrated a more than 2-fold increase (Figure 3B). It has been shown that the VLCFA-containing PSs may mediate transbilayer coupling, potentially via interdigitation. Moreover, cholesterol-dependent crosslinking of outer-leaflet VLCFA-sphingolipids with inner-leaflet 18:0/18:1-PS drives PS clustering, enabling signal transduction across the membrane [61]. This functional activity of PSs is realized mainly on the PM, where the majority of PS molecules and especially their VLCFA molecular species are localized [62].

The endomembrane response in Col-0 proved to be imbalanced: in contrast to *ixr1-1*, isoxaben reduced the content of plastidic MGDGs and DGDGs in Col-0 (Figure 2B). This may reflect a diminished role of endomembranes in the adaptation. Furthermore, the genotype-specific response (cluster I-A3, Figure 6) included an increase in the level of minor PEs containing VLCFAs. An increase in the concentration of these molecules together with VLCFA-PSs in the PM makes it thicker, facilitates interdigitation, affects interactions between layers, changes elasticity, and increases sensitivity to mechanical stress [61]. Thus, Col-0 prioritizes stress-induced stabilization and membrane structuring (via VLCFA-containing PEs and PSs, as well as PA signaling), which could be non-optimal for supporting the rapid hypocotyl elongation.

### 3.5. Mutant ixr1-1 Strategy: Regulating CSC Mobility via Global Membrane Disordering

The *ixr1-1* mutation not only provides resistance to isoxaben. It also confers reduced cellulose crystallinity [34], which may have consequences for mechanosensing at the cell wall level.

At the lipid class level, comparison of Col-0 and *ixr1-1* revealed a lower PC/PE ratio in the mutant due to the increased PE content (Figure 2A). This finding is physiologically significant because PCs form liquid-ordered microdomains, whereas PEs form liquid-disordered regions with reduced packing density and increased fluidity, creating packing defects important for membrane proteins [6]. Modulation of PC and PE relative content in membranes is one of the primary ways to regulate fluidity [7]. It is likely that the PM enrichment with liquid-disordered PEs increases CSC mobility. This assumption is supported by data on a closely related mutant, *ixr1-2*, in which reduced cellulose crystallinity correlated with increased CSC mobility [23].

Under isoxaben action, the relative content of PEs in *ixr1-1* mutant cells increases even more. Apparently, initially looser and prone to increased curvature due to the high content of PEs, *ixr1-1* membranes become even more disordered under the action of the inhibitor. A change in the PM structural organization resulting from an altered PC/PE ratio can have different consequences, both for membrane morphology and functionality. The PE-enriched membranes have been assumed to become more jagged and wavy, which should affect their contacts with the cell wall, for example, by increasing the contact area. In a study on soybean lines [63], isoxaben smoothed the sinuous edge of the PM in sensitive lines but made it more jagged in stable, resistant lines. This response could be associated with a change in the PC/PE ratio that modified the PM morphology and its contacts with the cell wall. Furthermore, a decrease in PC/PE can induce ER stress [64], which in plants triggers the maintenance of GPL homeostasis through the regulation of PCs and the enzymes of phosphoinositide metabolism [65,66]. In *ixr1-1*, the change in PC/PE correlated with a more stable profile of major molecular species of all studied lipid classes under isoxaben action (Figure 3A,B and Figure 4), suggesting that the *ixr1-1* resistance could be partly due to activation of lipid homeostasis mechanisms triggered by the altered PC/PE ratio.

PSs also play a critical role. The PS accumulation gradient is thought to be critically important for cell growth, in particular for the auxin signaling pathway on the PM, which is regulated by small RHO-OF-PLANTS6 GTPases. PSs stabilize these GTPases on the PM [66]. At the same time, the number of PS molecules can positively correlate with growth activity [67]. Our results showed that the *ixr1-1* mutant, which has a slightly higher PS content, retains it even upon isoxaben exposure (Figure 2A). Nevertheless, in contrast to Col-0, the profile of major molecular species of PSs remains unchanged (Figure 3B). It is worth noting that the similar stability in the relative content of major molecular species was characteristic for the two dominant GPL classes, including PCs and PEs, as well as PAs and PIs. However, the comprehensive analysis of GPL molecular species, including minor ones, still revealed a number of important features that clearly distinguish the lipidomes of the two genotypes and their response to isoxaben (Figure 5). The feature of *ixr1-1* is a higher content of PEs and PSs, also mostly with a saturated fatty acid. Interestingly, a significant part of them are odd-chain molecular species (with 15:0 and 17:0 fatty acids) and mixed-chain highly asymmetric species (14/18, 16/20, 18/22) (Figure 6). The accumulation of odd-chain and highly asymmetric molecular species in PEs deserves great attention. According to FTIR and ^2^H-NMR spectroscopy, the conformational and orientational order in the liquid-crystalline states of highly asymmetric-chain lipids differs markedly from that of comparable symmetric-chain lipids [68]. Moreover, the unusual shape of these lipids promotes interdigitation of hydrocarbon chains into the opposing monolayer [69]. These properties can significantly modify membrane characteristics, even when such GPL molecular species are present at low abundance. In particular, mixed-chain PCs have been shown to cause disorder in one or both leaflets [70]. Molecular species bearing odd-chain fatty acids demonstrated comparable effects, resulting in the disruption and disordering of the phospholipid bilayer [71].

Thus, lipidomics data, together with quantitative analysis at the lipid class level, indicate peculiarities of the physico-chemical properties of *ixr1-1* mutant membranes. All of them contribute to increased fluidity, lability and interdigitation between monolayers. In the PM, these properties probably alter the lipid microenvironment of CSCs, affecting their mobility and activity, which supports the isoxaben resistance. This systemic response also involves endomembranes: clusters II-A-1 and II-A-2, which include molecular species whose content is higher in the mutant, contained not only PEs but also plastidic MGDGs and DGDGs. Under stress conditions, GGLs, mainly DGDGs, can replace GPLs through their transport by carrier proteins and membrane contacts to other endomembranes, as well as to the PM [15]. These changes may affect the action of membrane proteins, in particular CSCs.

### 3.6. Genotype-Independent Universal Responses: Steady-State and Common Stress Lipidomes

Some isoxaben-induced lipid changes occurred in both Col-0 and *ixr1-1*, reflecting fundamental mechanisms that might not be mediated by the inhibitor binding to CSCs. These universal responses can be divided into two categories.

The steady-state lipidome (cluster II-B, Figure 6) is located at the bottom of the heat map and was particularly notable. It included molecular species whose levels were higher in both Col-0 and *ixr1-1* in the absence of isoxaben but decreased upon the inhibitor treatment. This “steady-state lipidome” [51] comprised major PC species, such as PC 16:0_18:2 and PC 18:2_18:2, as well as unsaturated PE and PS species. Furthermore, it was significantly enriched with 16/16 and 16/18 molecular species, including PC 16:3_18:4. This cluster likely represents the basal membrane composition under optimal growth conditions, reflecting the fundamental lipid requirements for normal cell function.

The universal isoxaben-response lipidome (cluster I-B, Figure 6) includes species that accumulated upon the inhibitor treatment regardless of genotype. It consists mainly of saturated (with one saturated fatty acid) and VLCFA-containing molecular types of PEs, as well as several PI and PA species with one saturated and one unsaturated fatty acid. The presence of these species in both Col-0 and *ixr1-1* points to signaling pathways that are likely activated independently of isoxaben interaction with CSCs. They could reflect common stress and mechanosensory mechanisms triggered by any challenge to the cell wall. PA accumulation, as noted above, is a universal “alarm” signal that enhances transmembrane transport and signal transmission [57]. The involvement of PI species is particularly interesting because phosphoinositides are key regulators of membrane signaling and cytoskeletal dynamics. Changes in PI profiles can affect the activity of phospholipase C and the production of inositol trisphosphate and diacylglycerol, both of which are important second messengers.

The existence of these universal responses has important implications. First, they demonstrate that even without direct binding to CSCs (as in *ixr1-1*), isoxaben still triggers a significant lipid remodeling program, likely through alternative binding sites or indirect mechanical signals from the altered cell wall. Second, the universal increase in VLCFA-containing PE species suggests that membrane thickening and increased interdigitation [61] establish a common adaptive strategy to cope with cell wall stress, regardless of genotype. Third, the presence of both steady-state and stress-induced universal lipidomes provides a baseline against which genotype-specific responses can be compared.

## 4. Materials and Methods

### 4.1. Plant Material and Growth Conditions

All experiments were carried out with four-day-old etiolated Col-0 wild-type or *ixr1-1* mutant seedlings of *Arabidopsis thaliana* (L. Heynh.) grown on vertical square Petri plates (120 mm × 120 mm × 17 mm, Greiner Bio-One, Mosonmagyaróvár, Hungary) under sterile conditions in solidified half-strength Murashige–Skoog medium containing 0.68% (*w*/*v*) micro-agar and no sucrose, as described in detail in [72]. Isoxaben (SC-235431, Santa Cruz Biotechnology, Inc., Dallas, TX, USA) was prepared as a 500 nM stock in DMSO and diluted to the intended concentration in hot (40 °C) growth medium before it was dispensed to the Petri plates.

### 4.2. Seedling Measurements

Close-up digital images of plants were taken with a Sony A7R3 digital mirrorless camera (Sony Inc., Tokyo, Japan) with a Canon EF 180 mm ƒ/3.5L Macro USM lens (Canon Inc., Tokyo, Japan) mounted via a Metabones Canon EF to a Sony E Mount T smart adapter or by 2× lens of Xiaomi 11 Lite, then geometry-corrected and color-corrected using an X-Rite Colorchecker Passport reference card (X-Rite Inc., Grand Rapids, MI, USA) in Adobe Camera RAW (Adobe Inc., San Jose, CA, USA). Images were processed in ImageJ (version 1.52p) for morphometry. Hypocotyl length was measured by tracing it from the root collar along the hypocotyl to the apical hook using a “segmented line” tool. The same approach was applied to measure cotyledon length, but using a “straight line” tool. At least 40 seedlings were examined for each experimental condition.

### 4.3. Plant Sampling and Lipid Extraction

Four-day-old seedlings of *A. thaliana* (approximately 160 seedlings per one biological replicate) were removed from the nutrient medium and cut into roots and shoots (hypocotyls with cotyledons). Excised shoots were extracted for lipids.

The plant material was homogenized in a mortar with 3 mL of chloroform: methanol 1:2 (*v*/*v*) mixture, and samples were incubated in glass tubes [73] at 4 °C overnight. Next, the samples were centrifuged to remove cell debris, 1 mL chloroform and 1.5 mL 2.5% (*w*/*v*) NaCl were added for phase separation. The bottom chloroform fraction containing total lipids was collected, and samples were dried in IKA rotary evaporator (Ika, Staufen, Germany).

### 4.4. Separation of Lipid Classes by TLC

Lipid extracts were separated by two-dimensional thin-layer chromatography (TLC) on silica gel 60 10 × 10 cm plates (Merck, Darmstadt, Germany). The plates were developed in a solvent system chloroform:methanol:water (65:25:4) in the first direction and in chloroform:acetone:methanol:acetic acid:water (50:20:10:10:5) in the second direction [74]. After temporary visualization in iodine vapors, lipid spots were scraped from TLC plates and eluted with chloroform:methanol (1:2) at 4 °C overnight. The resulting eluates were then centrifuged to remove the silica gel, the supernatant was evaporated, and the lipids were redissolved in 40 µL of HPLC-grade methanol in 1.5 mL vials. Quality control samples were prepared by combining 5 μL of each sample extract.

Quantification of lipid classes was determined densitometrically using a Denscan (Lenchrom, Russia). Phosphatidylcholine (PC) from egg yolk (Sigma-Aldrich, St. Louis, MO, USA) and monogalactosyldiacylglycerol (MGDG) (Avanti Polar Lipids, Alabaster, AL, USA) were used as standards. The number of analytical replicates for densitometric analysis was two per sample.

### 4.5. Lipid Profiling

Lipid profiles were analyzed by LC-MS/MS using a LCMS-8030 triple quadrupole mass spectrometer (Shimadzu, Kyoto, Japan) with a Nexera UHPLC system (Shimadzu, Kyoto, Japan). Samples were injected automatically with an SIL-30AC autosampler onto a Kinetex C18 column (2.6 µm, 2.1 × 150 mm, 100 Å; Phenomenex, Torrance, CA, USA); the autosampler was set to 4 °C. The flow rate was 0.3 mL/min, and the column oven temperature was set at 50 °C. Mobile phases were acetonitrile/water 1:1 (*v*/*v*) as a solvent A and 2-propanol/acetonitrile/water 85:10:5 (*v*/*v*/*v*) as a solvent B, both containing 5 mM ammonium formate and 0.1% (*v*/*v*) formic acid [75]. The gradient program was as follows: 1 min, 45% B; 11 min, 90% B; 11.1 min, 100% B; 15 min, 100% B; 15.1 min, 45% B; 16 min, 45% B (stop time). The following parameters were used for ionization: nebulizing gas flow 3.0 L/min, drying gas flow 15.0 L/min, DL temperature 250 °C, heat block temperature 400 °C; ESI interface voltage, 4500 V (+), 3500 V (–). The number of injections (analytical replicates) typically did not exceed one per sample. In some cases, the analysis was repeated to exclude technical outliers.

A two-step MS/MS approach was used for lipid profiling. For the untargeted step, a precursor ion scan for PC and PI and a neutral loss scan for PE, PS, PG, MGDG, and DGDG were used to screen for functional groups associated with particular lipid classes. PC profiling was performed in a positive ion mode by monitoring the product ion of m/z 184.07. PI was profiled in a negative ion mode using the precursor ion scan of m/z 241.01. Collision energy was set as −33 V for PC and −44 V for PI. Profiles of PE, PS, PG, MGDG, and DGDG were performed in a positive ion mode using the neutral loss scan of m/z 141.01 (CE -35), 185.01 (CE -25), 189 (CE -25), 179.1 (CE -15), and 341.1 (CE -18), respectively. The parameters were set as follows: MS1 mass range, 600–1050 m/z, event time, 100 ms; Q1 resolution, high; Q3 resolution, unit. Based on the data of this untargeted approach, a panel of phospholipid multiple reaction monitoring (MRM) transitions was prepared using detected molecular masses of lipids and theoretical values of fatty acid-related fragmentation for more accurate quantification. MRM parameters: dwell time, 5 ms; Q1 and Q3 resolution, unit.

A blank control and three quality control (QC) samples were interspersed after every seven experimental samples to ensure the quality of the batch. Peak areas of each molecular species were normalized to the absolute amount of lipid class quantified densitometrically. The mixture of PC 13:0/13:0, PC 17:0/17:0, PC 19:0/19:0, PE 15:0/15:0, PE 16:0/16:0, PG 14:0/14:0, PG 16:0/16:0, and PG 18:0/18:0 molecular species was used as an external control to prove that the signal intensity reflected the relative abundance of the phospholipid molecular species under the conditions used. PC, PE, PG molecular species were from Avanti Polar Lipids (Alabaster, Montgomery, AL, USA).

Data were processed in LabSolutions Postrun Analysis 5.98SP1 (Shimadzu) and Skyline [76].

### 4.6. Data Analysis and Visualization

All experiments were carried out in at least three biological replicates. Statistical analysis and visualization were performed in Microsoft Excel 16.0 (Microsoft, Redmond, WA, USA) and R 4.3.1 “Beagle Scouts” [77]. A Shapiro–Wilk test was used to check for normal distribution. A Mann–Whitney test was performed as a non-parametric test. A Benjamini–Hochberg test was carried out for p-adjustments. ANOVA combined with a Tukey post hoc test was performed for parametric comparisons. Data on histograms are means with standard deviations. Morphometric data were filtered by the Hampel method.

Lipidomic data preprocessing included outliers detection and exclusion on the basis of Grubb’s test. If the value was excluded or was zero, while it was non-zero in other replications, it was postulated as a randomly missed value and was imputed with the KNN (k-nearest neighbors) method from “impute” R package [77]. Principal component analysis (PCA) from the R-package “pcaMethods” [78] was used for statistical analysis of lipid profiles. Orthogonal partial least square discriminant analysis (OPLS-DA) was performed in the “ropls” package. VIP (variable importance in projection) values were used for feature selection [79]. Heat maps combined with hierarchical clustering were created by in-house code using functions from the packages “base”, “stats” [77] and “spiralize” [80]. Hierarchical clustering was made by the Ward method using Spearman’s distance (1-r, where r is Spearman’s rank correlation).

## 5. Conclusions

We studied lipidomes of etiolated *A. thaliana* hypocotyls to reveal changes in the PM and endomembrane lipids associated with cellulose synthesis modulation. Using Col-0 wild-type and *ixr1-1* mutant plants resistant to isoxaben, grown with or without this specific cellulose biosynthesis inhibitor, we detected changes in the main classes of phospholipids, glycolipids and their 250 molecular species. Two lipidome-level strategies of response to isoxaben have been established.

The first strategy in Col-0, where isoxaben severely suppressed growth, involved fine remodeling of membrane GPLs with an increase in the absolute and/or relative content of unsaturated molecular species of PCs and PAs, such as 16:1_18:3, 18:1_18:3, 18:3_18:3, and simultaneously an increase in VLCFA-containing PEs and VLCFA-containing PSs, such as 18:2_22:0 and 18:2_24:0. This strategy is associated with a decrease in thickness and an increase in disorder of the outer leaflet, and the opposite effect on the inner leaflet of membranes. Col-0 also demonstrated signs of a stress response, including a three-fold increase in PA content and a significant reduction in endomembrane lipids, including MGDGs and DGDGs.

The second strategy in the mutant, where hypocotyl growth was not inhibited by isoxaben, involved reorganization at the level of lipid classes: the PC/PE ratio decreased due to active accumulation of PEs, and the contribution of endomembrane lipids, including MGDGs, DGDGs, and DPGs, significantly increased. Unlike Col-0, the profile of major lipid molecular species remained highly stable in *ixr1-1*; however, an increase in the content of minor PEs with atypical molecular species (odd-chain species, highly asymmetric chain-length species) was observed. All these changes in *ixr1-1* potentially increase membrane fluidity and disorder, rendering them more labile.

Our findings demonstrate the importance of lipid environment for CSC function, which, by modulating membrane thickness, fluidity, order, charge, and other characteristics, can regulate CSC mobility, position, and thereby enzymatic activity.

## Data Availability

The data presented in this study are available on request from the corresponding author. The data are not publicly available due to privacy restrictions.

## Supplementary Materials

The following supporting information can be downloaded at: https://www.mdpi.com/article/10.3390/ijms27125424/s1.

## Abbreviations/Acronyms

CSC	Cellulose Synthase Complex
DGDG(s)	Digalactosyldiacylglycerol(s)
DPG(s)	Diphosphatidylglycerol(s)
GGL(s)	Galactoglycerolipids(s)
GPLs	Glycerophospholipids
ISX	Isoxaben
LC-QqQ-MS/MS	Liquid Chromatography–Triple Quadrupole Tandem Mass Spectrometry
MDS	Multidimensional Scaling
MGDG(s)	Monogalactosyldiacylglycerol(s)
MRM	Multiple Reaction Monitoring Method
OPLS-DA	Orthogonal Partial Least Squares-Discriminant Analysis
PA(s)	Phosphatidic Acid(s)
PC(s)	Phosphatidylcholine(s)
PCA	Principal Component Analysis
PE(s)	Phosphatidylethanolamine(s)
PG(s)	Phosphatidylglycerol(s)
PI(s)	Phosphatidylinositol(s)
PM	Plasma Membrane
PS(s)	Phosphatidylserine(s)
SQDG(s)	Sulfoquinovosyldiacylglycerol(s)
TLC	Thin-Layer Chromatography
VLCFA(s)	Very-Long-Chain Fatty Acid(s)

## References

[1] Nakamura Y. (2017). Plant Phospholipid Diversity: Emerging Functions in Metabolism and Protein-Lipid Interactions. Trends Plant Sci..

[2] Harayama T., Riezman H. (2018). Understanding the Diversity of Membrane Lipid Composition. Nat. Rev. Mol. Cell Biol..

[3] Barrero-Sicilia C., Silvestre S., Haslam R.P., Michaelson L.V. (2017). Lipid Remodelling: Unravelling the Response to Cold Stress in Arabidopsis and Its Extremophile Relative Eutrema Salsugineum. Plant Sci..

[4] Nakamura Y. (2018). Membrane Lipid Oscillation: An Emerging System of Molecular Dynamics in the Plant Membrane. Plant Cell Physiol..

[5] Kehelpannala C., Rupasinghe T., Pasha A., Esteban E., Hennessy T., Bradley D., Ebert B., Provart N.J., Roessner U. (2021). An Arabidopsis Lipid Map Reveals Differences between Tissues and Dynamic Changes throughout Development. Plant J..

[6] Casares D., Escribá P.V., Rosselló C.A. (2019). Membrane Lipid Composition: Effect on Membrane and Organelle Structure, Function and Compartmentalization and Therapeutic Avenues. Int. J. Mol. Sci..

[7] De Mendoza D., Pilon M. (2019). Control of Membrane Lipid Homeostasis by Lipid-Bilayer Associated Sensors: A Mechanism Conserved from Bacteria to Humans. Prog. Lipid Res..

[8] Marković V., Jaillais Y. (2022). Phosphatidylinositol 4-phosphate: A Key Determinant of Plasma Membrane Identity and Function in Plants. New Phytol..

[9] Goldy C., Caillaud M.-C. (2023). Connecting the Plant Cytoskeleton to the Cell Surface via the Phosphoinositides. Curr. Opin. Plant Biol..

[10] Zhou H., Huo Y., Yang N., Wei T. (2024). Phosphatidic Acid: From Biophysical Properties to Diverse Functions. FEBS J..

[11] Holthuis J.C.M., Menon A.K. (2014). Lipid Landscapes and Pipelines in Membrane Homeostasis. Nature.

[12] Corradi V., Sejdiu B.I., Mesa-Galloso H., Abdizadeh H., Noskov S.Y., Marrink S.J., Tieleman D.P. (2019). Emerging Diversity in Lipid–Protein Interactions. Chem. Rev..

[13] Noack L.C., Jaillais Y. (2020). Functions of Anionic Lipids in Plants. Annu. Rev. Plant Biol..

[14] Yoshihara A., Nagata N., Wada H., Kobayashi K. (2021). Plastid Anionic Lipids Are Essential for the Development of Both Photosynthetic and Non-Photosynthetic Organs in Arabidopsis Thaliana. Int. J. Mol. Sci..

[15] Hölzl G., Dörmann P. (2019). Chloroplast Lipids and Their Biosynthesis. Annu. Rev. Plant Biol..

[16] Lagace T.A., Ridgway N.D. (2013). The Role of Phospholipids in the Biological Activity and Structure of the Endoplasmic Reticulum. Biochim. Biophys. Acta BBA-Mol. Cell Res..

[17] Smith C.N., Haslam T.M., Feussner I., Roston R.L. (2026). Subcellular Lipid Trafficking and Membrane Specialization in Plants. Annu. Rev. Plant Biol..

[18] Baskin T.I. (2005). Anisotropic expansion of the plant cell wall. Annu. Rev. Cell Dev. Biol..

[19] Zhang Y., Nikolovski N., Sorieul M., Vellosillo T., McFarlane H.E., Dupree R., Kesten C., Schneider R., Driemeier C., Lathe R. (2016). Golgi-Localized STELLO Proteins Regulate the Assembly and Trafficking of Cellulose Synthase Complexes in Arabidopsis. Nat. Commun..

[20] He M., Lan M., Zhang B., Zhou Y., Wang Y., Zhu L., Yuan M., Fu Y. (2018). Rab-H1b Is Essential for Trafficking of Cellulose Synthase and for Hypocotyl Growth in Arabidopsis Thaliana. J. Integr. Plant Biol..

[21] Pedersen G.B., Blaschek L., Frandsen K.E.H., Noack L.C., Persson S. (2023). Cellulose Synthesis in Land Plants. Mol. Plant.

[22] Ivakov A., Flis A., Apelt F., Fünfgeld M., Scherer U., Stitt M., Kragler F., Vissenberg K., Persson S., Suslov D. (2017). Cellulose Synthesis and Cell Expansion Are Regulated by Different Mechanisms in Growing Arabidopsis Hypocotyls. Plant Cell.

[23] Harris D.M., Corbin K., Wang T., Gutierrez R., Bertolo A.L., Petti C., Smilgies D.-M., Estevez J.M., Bonetta D., Urbanowicz B.R. (2012). Cellulose Microfibril Crystallinity Is Reduced by Mutating C-Terminal Transmembrane Region Residues CESA1A903V and CESA3T942I of Cellulose Synthase. Proc. Natl. Acad. Sci. USA.

[24] Schrick K., Fujioka S., Takatsuto S., Stierhof Y., Stransky H., Yoshida S., Jürgens G. (2004). A Link between Sterol Biosynthesis, the Cell Wall, and Cellulose in Arabidopsis. Plant J..

[25] Fang L., Ishikawa T., Rennie E.A., Murawska G.M., Lao J., Yan J., Tsai A.Y.-L., Baidoo E.E.K., Xu J., Keasling J.D. (2016). Loss of Inositol Phosphorylceramide Sphingolipid Mannosylation Induces Plant Immune Responses and Reduces Cellulose Content in Arabidopsis. Plant Cell.

[26] Li M., Bahn S.C., Guo L., Musgrave W., Berg H., Welti R., Wang X. (2011). Patatin-Related Phospholipase pPLAIIIβ-Induced Changes in Lipid Metabolism Alter Cellulose Content and Cell Elongation in Arabidopsis. Plant Cell.

[27] Zheng Y., Li M., Wang X. (2014). Proteomic Insight into Reduced Cell Elongation Resulting from Overexpression of Patatin-Related Phospholipase pPLAIIIδ in Arabidopsis Thaliana. Plant Signal. Behav..

[28] Jang J.H., Seo H.S., Lee O.R. (2021). The Reduced Longitudinal Growth Induced by Overexpression of pPLAIIIγ Is Regulated by Genes Encoding Microtubule-Associated Proteins. Plants.

[29] Huang L., Li X., Zhang C. (2021). Endosidin20-1 Is More Potent than Endosidin20 in Inhibiting Plant Cellulose Biosynthesis and Molecular Docking Analysis of Cellulose Biosynthesis Inhibitors on Modeled Cellulose Synthase Structure. Plant J..

[30] Ogden M., Whitcomb S.J., Khan G.A., Roessner U., Hoefgen R., Persson S. (2024). Cellulose Biosynthesis Inhibitor Isoxaben Causes Nutrient-Dependent and Tissue-Specific Arabidopsis Phenotypes. Plant Physiol..

[31] DeBolt S., Gutierrez R., Ehrhardt D.W., Somerville C. (2007). Nonmotile Cellulose Synthase Subunits Repeatedly Accumulate within Localized Regions at the Plasma Membrane in Arabidopsis Hypocotyl Cells Following 2,6-Dichlorobenzonitrile Treatment. Plant Physiol..

[32] Tran M.L., McCarthy T.W., Sun H., Wu S.-Z., Norris J.H., Bezanilla M., Vidali L., Anderson C.T., Roberts A.W. (2018). Direct Observation of the Effects of Cellulose Synthesis Inhibitors Using Live Cell Imaging of Cellulose Synthase (CESA) in Physcomitrella Patens. Sci. Rep..

[33] Heim D.R., Roberts J.L., Pike P.D., Larrinua I.M. (1989). Mutation of a Locus of Arabidopsis Thaliana Confers Resistance to the Herbicide Isoxaben. Plant Physiol..

[34] Scheible W.-R., Eshed R., Richmond T., Delmer D., Somerville C. (2001). Modifications of Cellulose Synthase Confer Resistance to Isoxaben and Thiazolidinone Herbicides in Arabidopsis Ixr1 Mutants. Proc. Natl. Acad. Sci. USA.

[35] Shim I., Law R., Kileeg Z., Stronghill P., Northey J.G.B., Strap J.L., Bonetta D.T. (2018). Alleles Causing Resistance to Isoxaben and Flupoxam Highlight the Significance of Transmembrane Domains for CESA Protein Function. Front. Plant Sci..

[36] Gendreau E., Traas J., Desnos T., Grandjean O., Caboche M., Hofte H. (1997). Cellular Basis of Hypocotyl Growth in Arabidopsis Thaliana. Plant Physiol..

[37] Refregier G., Pelletier S., Jaillard D., Höfte H. (2004). Interaction between Wall Deposition and Cell Elongation in Dark-Grown Hypocotyl Cells in Arabidopsis. Plant Physiol..

[38] Suslov D., Ivakov A., Boron A.K., Vissenberg K. (2015). In Vitro Cell Wall Extensibility Controls Age-Related Changes in the Growth Rate of Etiolated Arabidopsis Hypocotyls. Funct. Plant Biol..

[39] De Kroon A.I.P.M. (2017). Lipidomics in Research on Yeast Membrane Lipid Homeostasis. Biochim. Biophys. Acta BBA-Mol. Cell Biol. Lipids.

[40] Kimura T., Jennings W., Epand R.M. (2016). Roles of Specific Lipid Species in the Cell and Their Molecular Mechanism. Prog. Lipid Res..

[41] Levental I., Lyman E. (2023). Regulation of Membrane Protein Structure and Function by Their Lipid Nano-Environment. Nat. Rev. Mol. Cell Biol..

[42] Wanner G., Schroeder-Reiter E., Assaad F.F. (2025). Cryo-SEM and Large Volume FIB-SEM of Arabidopsis Cotyledons: Degradation of Lipid Bodies, Biogenesis of Glyoxysomes and Reorganisation of Organelles during Germination. J. Microsc..

[43] Cosgrove D.J. (2016). Catalysts of Plant Cell Wall Loosening. F1000Research.

[44] Polko J.K., Kieber J.J. (2019). The Regulation of Cellulose Biosynthesis in Plants. Plant Cell.

[45] Baez L.A., Tichá T., Hamann T. (2022). Cell Wall Integrity Regulation across Plant Species. Plant Mol. Biol..

[46] Pilling E., Höfte H. (2003). Feedback from the Wall. Curr. Opin. Plant Biol..

[47] Merz D., Richter J., Gonneau M., Sanchez-Rodriguez C., Eder T., Sormani R., Martin M., Hématy K., Höfte H., Hauser M.-T. (2017). T-DNA Alleles of the Receptor Kinase THESEUS1 with Opposing Effects on Cell Wall Integrity Signaling. J. Exp. Bot..

[48] Worden N., Wilkop T.E., Esteve V.E., Jeannotte R., Lathe R., Vernhettes S., Weimer B., Hicks G., Alonso J., Labavitch J. (2015). CESA TRAFFICKING INHIBITOR Inhibits Cellulose Deposition and Interferes with the Trafficking of Cellulose Synthase Complexes and Their Associated Proteins KORRIGAN1 and POM2/CELLULOSE SYNTHASE INTERACTIVE PROTEIN1. Plant Physiol..

[49] Renou J., Li D., Lu J., Zhang B., Gineau E., Ye Y., Shi J., Voxeur A., Akary E., Marmagne A. (2024). A Cellulose Synthesis Inhibitor Affects Cellulose Synthase Complex Secretion and Cortical Microtubule Dynamics. Plant Physiol..

[50] Nakagawa N., Sakurai N. (2006). A Mutation in At-nMat1a, Which Encodes a Nuclear Gene Having High Similarity to Group II Intron Maturase, Causes Impaired Splicing of Mitochondrial NAD4 Transcript and Altered Carbon Metabolism in Arabidopsisthaliana. Plant Cell Physiol..

[51] Hu Z., Vanderhaeghen R., Cools T., Wang Y., De Clercq I., Leroux O., Nguyen L., Belt K., Millar A.H., Audenaert D. (2016). Mitochondrial Defects Confer Tolerance against Cellulose Deficiency. Plant Cell.

[52] Awwad F., Bertrand G., Grandbois M., Beaudoin N. (2019). Auxin Protects Arabidopsis Thaliana Cell Suspension Cultures from Programmed Cell Death Induced by the Cellulose Biosynthesis Inhibitors Thaxtomin A and Isoxaben. BMC Plant Biol..

[53] Allagulova C.R., Lubyanova A.R., Avalbaev A.M. (2023). Multiple Ways of Nitric Oxide Production in Plants and Its Functional Activity under Abiotic Stress Conditions. Int. J. Mol. Sci..

[54] Zhao L., Liu F., Crawford N.M., Wang Y. (2018). Molecular Regulation of Nitrate Responses in Plants. Int. J. Mol. Sci..

[55] Nakamura Y., Ngo A.H. (2020). Non-Specific Phospholipase C (NPC): An Emerging Class of Phospholipase C in Plant Growth and Development. J. Plant Res..

[56] Maatta S., Scheu B., Roth M.R., Tamura P., Li M., Williams T.D., Wang X., Welti R. (2012). Levels of Arabidopsis Thaliana Leaf Phosphatidic Acids, Phosphatidylserines, and Most Trienoate-Containing Polar Lipid Molecular Species Increase during the Dark Period of the Diurnal Cycle. Front. Plant Sci..

[57] Pokotylo I., Kravets V., Martinec J., Ruelland E. (2018). The Phosphatidic Acid Paradox: Too Many Actions for One Molecule Class? Lessons from Plants. Prog. Lipid Res..

[58] Bates P.D., Browse J. (2011). The Pathway of Triacylglycerol Synthesis through Phosphatidylcholine in Arabidopsis Produces a Bottleneck for the Accumulation of Unusual Fatty Acids in Transgenic Seeds. Plant J..

[59] Hoffmann D.Y., Shachar-Hill Y. (2023). Do Betaine Lipids Replace Phosphatidylcholine as Fatty Acid Editing Hubs in Microalgae?. Front. Plant Sci..

[60] Kotlova E.R., Senik S.V., Pozhvanov G.A., Prokopiev I.A., Boldyrev I.A., Manzhieva B.S., Amigud E.Y., Puzanskiy R.K., Khakulova A.A., Serebryakov E.B. (2023). Uptake and Metabolic Conversion of Exogenous Phosphatidylcholines Depending on Their Acyl Chain Structure in Arabidopsis Thaliana. Int. J. Mol. Sci..

[61] Batsale M., Bahammou D., Fouillen L., Mongrand S., Joubès J., Domergue F. (2021). Biosynthesis and Functions of Very-Long-Chain Fatty Acids in the Responses of Plants to Abiotic and Biotic Stresses. Cells.

[62] Colin L.A., Jaillais Y. (2020). Phospholipids across Scales: Lipid Patterns and Plant Development. Curr. Opin. Plant Biol..

[63] Corio-Costet M.-F., Lherminier J., Scalla R. (1991). Effects of Isoxaben on Sensitive and Tolerant Plant Cell Cultures. Pestic. Biochem. Physiol..

[64] Yu C.-Y., Nguyen V.C., Chuang L., Kanehara K. (2018). Membrane Glycerolipid Equilibrium under Endoplasmic Reticulum Stress in Arabidopsis Thaliana. Biochem. Biophys. Res. Commun..

[65] Shank K.J., Su P., Brglez I., Boss W.F., Dewey R.E., Boston R.S. (2001). Induction of Lipid Metabolic Enzymes during the Endoplasmic Reticulum Stress Response in Plants. Plant Physiol..

[66] Platre M.P., Bayle V., Armengot L., Bareille J., Marquès-Bueno M.D.M., Creff A., Maneta-Peyret L., Fiche J.-B., Nollmann M., Miège C. (2019). Developmental Control of Plant Rho GTPase Nano-Organization by the Lipid Phosphatidylserine. Science.

[67] Dubois G.A., Jaillais Y. (2021). Anionic Phospholipid Gradients: An Uncharacterized Frontier of the Plant Endomembrane Network. Plant Physiol..

[68] Lewis R.N., McElhaney R.N., Monck M.A., Cullis P.R. (1994). Studies of Highly Asymmetric Mixed-Chain Diacyl Phosphatidylcholines That Form Mixed-Interdigitated Gel Phases: Fourier Transform Infrared and 2H NMR Spectroscopic Studies of Hydrocarbon Chain Conformation and Orientational Order in the Liquid-Crystalline State. Biophys. J..

[69] Mattai J., Sripada P.K., Shipley G.G. (1987). Mixed-Chain Phosphatidylcholine Bilayers: Structure and Properties. Biochemistry.

[70] Frewein M.P.K., Piller P., Semeraro E.F., Batchu K.C., Heberle F.A., Scott H.L., Gerelli Y., Porcar L., Pabst G. (2022). Interdigitation-Induced Order and Disorder in Asymmetric Membranes. J. Membr. Biol..

[71] Yoon B.K., Tan S.W., Tan J.Y.B., Jackman J.A., Cho N.-J. (2022). Nanoarchitectonics-Based Model Membrane Platforms for Probing Membrane-Disruptive Interactions of Odd-Chain Antimicrobial Lipids. Nano Converg..

[72] Pozhvanov G., Suslov D. (2024). Sucrose and Mannans Affect Arabidopsis Shoot Gravitropism at the Cell Wall Level. Plants.

[73] Bligh E.G., Dyer W.J. (1959). A rapid method of total lipid extraction and purification. Can. J. Biochem. Physiol..

[74] Benning C., Huang Z.H., Gage D.A. (1995). Accumulation of a Novel Glycolipid and a Betaine Lipid in Cells of Rhodobacter Sphaeroides Grown under Phosphate Limitation. Arch. Biochem. Biophys..

[75] Lange M., Angelidou G., Ni Z., Criscuolo A., Schiller J., Blüher M., Fedorova M. (2021). AdipoAtlas: A Reference Lipidome for Human White Adipose Tissue. Cell Rep. Med..

[76] Adams K.J., Pratt B., Bose N., Dubois L.G., St. John-Williams L., Perrott K.M., Ky K., Kapahi P., Sharma V., MacCoss M.J. (2020). Skyline for Small Molecules: A Unifying Software Package for Quantitative Metabolomics. J. Proteome Res..

[77] R: The R Project for Statistical Computing. https://www.r-project.org/.

[78] Stacklies W., Redestig H., Scholz M., Walther D., Selbig J. (2007). pcaMethods—A Bioconductor Package Providing PCA Methods for Incomplete Data. Bioinformatics.

[79] Trevor Hastie R.T. (2017). Impute. Bioconductor.

[80] Gu Z., Hübschmann D. (2022). Spiralize: An R package for visualizing data on spirals. Bioinformatics.

