# Supplementary figures and images for: Plasma Membrane and Endomembrane Lipids Are Involved in a Complex Adaptation of Arabidopsis thaliana Hypocotyls to Cellulose Biosynthesis Inhibition
Source: Int J Mol Sci. 2026 Jun 16;27(12):5424. doi: 10.3390/ijms27125424 (PMC13299368; doi:10.3390/ijms27125424)

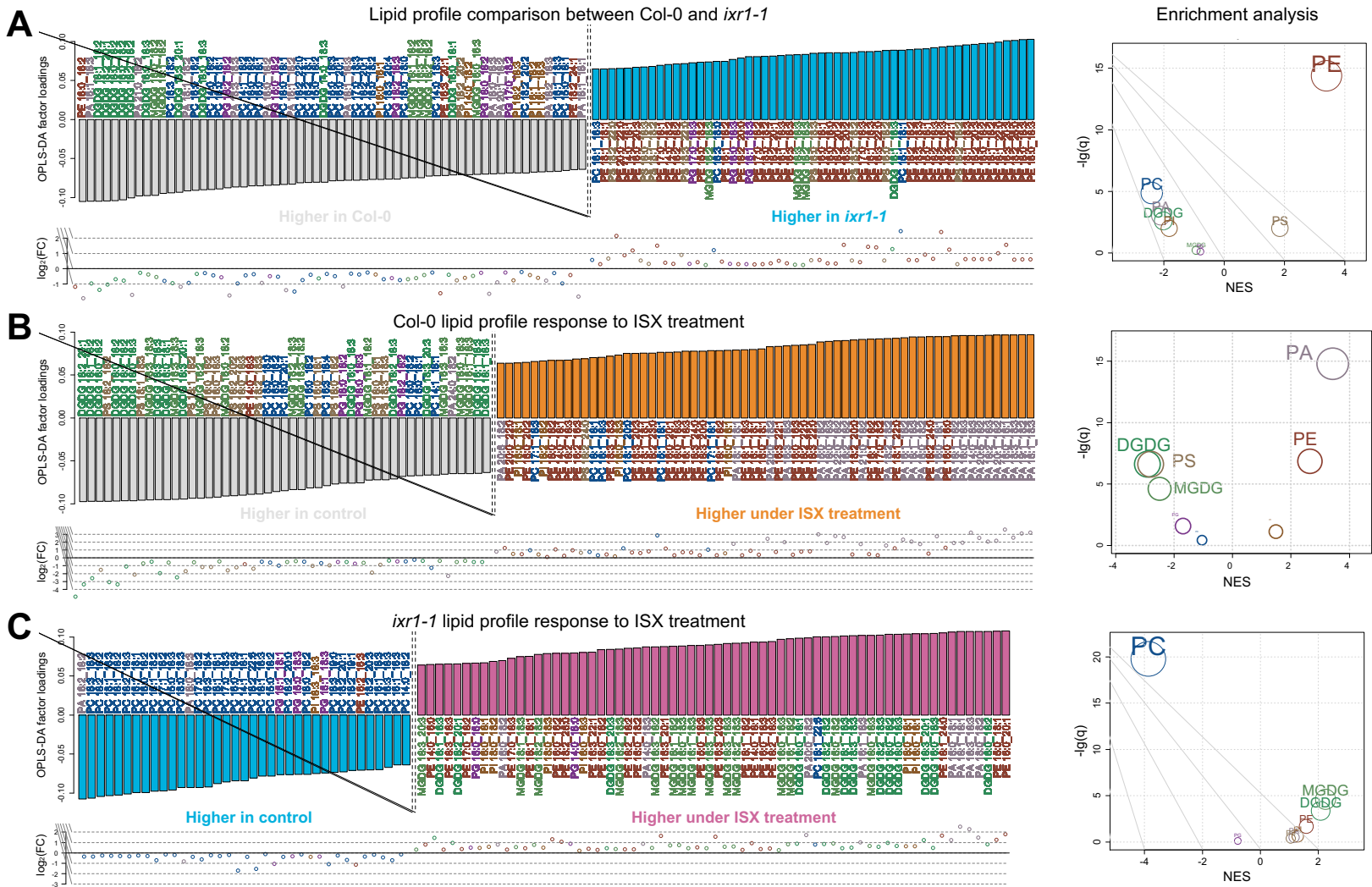

Supplement: Supplementary file 1 [file ijms-27-05424-s001.zip › Figure S1.pdf]
